# Relevance of TNBS-Colitis in Rats: A Methodological Study with Endoscopic, Histologic and Transcriptomic Characterization and Correlation to IBD

**DOI:** 10.1371/journal.pone.0054543

**Published:** 2013-01-31

**Authors:** Øystein Brenna, Marianne W. Furnes, Ignat Drozdov, Atle van Beelen Granlund, Arnar Flatberg, Arne K. Sandvik, Rosalie T. M. Zwiggelaar, Ronald Mårvik, Ivar S. Nordrum, Mark Kidd, Björn I. Gustafsson

**Affiliations:** 1 Department of Gastroenterology and Hepatology, St. Olavs Hospital, Trondheim University Hospital, Trondheim, Norway; 2 Department of Cancer Research and Molecular Medicine, Norwegian University of Science and Technology, Trondheim, Norway; 3 Bering Limited, Richmond, United Kingdom; 4 Department of Gastrointestinal Surgery, St. Olavs Hospital, Trondheim University Hospital, Trondheim, Norway; 5 Department of Pathology and Medical Genetics, St. Olavs Hospital, Trondheim University Hospital, Trondheim, Norway; 6 Department of Surgery, Section of Gastroenterology, Yale School of Medicine, New Haven, Connecticut, United States of America; Institut Pasteur de Lille, France

## Abstract

**Background:**

Rectal instillation of trinitrobenzene sulphonic acid (TNBS) in ethanol is an established model for inflammatory bowel disease (IBD). We aimed to 1) set up a TNBS-colitis protocol resulting in an endoscopic and histologic picture resembling IBD, 2) study the correlation between endoscopic, histologic and gene expression alterations at different time points after colitis induction, and 3) compare rat and human IBD mucosal transcriptomic data to evaluate whether TNBS-colitis is an appropriate model of IBD.

**Methodology/Principal Findings:**

Five female Sprague Daley rats received TNBS diluted in 50% ethanol (18 mg/0.6 ml) rectally. The rats underwent colonoscopy with biopsy at different time points. RNA was extracted from rat biopsies and microarray was performed. PCR and *in situ* hybridization (ISH) were done for validation of microarray results. Rat microarray profiles were compared to human IBD expression profiles (25 ulcerative colitis Endoscopic score demonstrated mild to moderate colitis after three and seven days, but declined after twelve days. Histologic changes corresponded with the endoscopic appearance. Over-represented Gene Ontology Biological Processes included: *Cell Adhesion*, *Immune Response*, *Lipid Metabolic Process*, and *Tissue Regeneration*. IL-1α, IL-1β, TLR2, TLR4, PRNP were all significantly up-regulated, while PPARγ was significantly down-regulated. Among genes with highest fold change (FC) were SPINK4, LBP, ADA, RETNLB and IL-1α. The highest concordance in differential expression between TNBS and IBD transcriptomes was three days after colitis induction. ISH and PCR results corresponded with the microarray data. The most concordantly expressed biologically relevant pathways included *TNF signaling*, *Cell junction organization*, and *Interleukin-1 processing*.

**Conclusions/Significance:**

Endoscopy with biopsies in TNBS-colitis is useful to follow temporal changes of inflammation visually and histologically, and to acquire tissue for gene expression analyses. TNBS-colitis is an appropriate model to study specific biological processes in IBD.

## Introduction

Inflammatory bowel disease (IBD) is the common denomination of ulcerative colitis (UC) and Crohn's disease (CD). The etiology is unknown and the pathogenesis is complex and incompletely understood. The interplay between genetic and immunological host factors and the gut microbiota are important factors in the development of disease [1], [2].

The inflammatory response in IBD is characterized by mucosal barrier dysfunction, microbial invasion and activation of immune response [3], [4]. In genetically predisposed individuals, microbial activation via toll-like receptors (TLRs) and induction of an inflammatory response accompanied by high levels of pro-inflammatory cytokines such as interleukins (ILs) (IL-1, IL-6, IL-13 and IL-17) and tumor necrosis factor alpha (TNF-α), seem to be critical [1], [5]–[9]. However, the exact molecular basis of IBD remains poorly understood.

Experimentally induced colitis with trinitrobenzene sulphonic acid (TNBS) is used to generate models that are used to examine the pathogenesis of gut inflammation, and determine the mechanisms and efficacy of therapies [10]. TNBS is diluted in ethanol which disrupts the mucosal barrier. Usually, the TNBS-solution is rectally instilled. Inflammation is induced by TNBS-induced haptenization of colonic mucosal proteins [11]. The use of TNBS to generate colitis was originally described and histologically characterized by Morris *et al*. in rats. Granulomas were observed in over 50% of animals two to three weeks after induction of colitis [12]. TNBS-ethanol administration is characterized by Th1-driven inflammation [11], and has primarily been regarded as a model for CD [10], [13]. The clinical features include weight loss and bloody diarrhea, while morphologically the model is characterized by mucosal, submucosal and transmural inflammation. Consequently, the model has been used to investigate the role and mechanistic action of drugs including 5-aminosalisylic acid, steroids and anti-tumor necrosis factor (TNF) in IBD [14]–[16]. The administration and doses of TNBS differ significantly between studies; the methodology is inconsistent and no standardized protocol exists [14], [17]–[20].

Both TNBS-colitis and IBD include disturbances in basic physiological processes like immune activation, metabolism and mucosal repair [21]. Microbial regulation of TLRs and induction of an inflammatory response is accompanied by regulation of pro- and anti-inflammatory cytokines and the shaping of the intestinal immune response [22]. Down-regulation of the peroxisome proliferator-activated receptor (PPAR) gamma involved in the regulation of fatty acid metabolism and inflammation, has been demonstrated in IBD and is associated with maintenance of defensin expression [23]–[25]. The cellular Prion protein (PrP^c^) is expressed in brain and various extra cerebral tissues including neuroendocrine cells and lymphoid tissue of the gut [26]–[28]. PrP^c^ can have an anti-inflammatory effect in the colon [29].

We report the development and characterization of TNBS-colitis in rats using colonoscopy and temporal gene expression profiling. The aim was to standardize TNBS administration to achieve a moderate inflammation. Achieving a moderate colitis allowed for the study of epithelial alterations upon mucosal damage, while temporal gene expression profiling identified longitudinal transcriptomic changes that were associated with these abnormalities. We further aimed to compare the genome-wide changes in TNBS-colitis to a human transcriptome to determine whether TNBS at this dose was an appropriate model for IBD.

## Materials and Methods

### Animals

The study was approved by the National Animal Research Authority (NARA). The general care and use of the animals were in accordance with the European Convention for the protection of Vertebrate Animals used for Experimental and other Scientific purposes. We used female Sprague Dawley rats weighing 200–250 g from Taconic (Taconic Farms, Inc., Hallingore, Denmark). They were housed in individually ventilated cages (IVC) with aspen bedding (Tapvei) in a specific pathogen free (SPF) environment with temperature 19–22°C and humidity 50–60% and 12 hr day and night cycle with 1 hr dusk and dawn. All rats had access to water and standard chow (RM1 maintenance, SDS, UK) *ad libitum*, apart from the 24 hr fasting periods before each colonoscopy to allow voiding of the left colon, when they had access only to glucose 2% fluid mixture and were kept in wired bottom cages. Rat colonoscopy was performed under general gas anesthesia using isoflurane. At termination, the rats were sacrificed by exsanguination during general isoflurane anesthesia.

### Initial studies and main study

#### Initial studies to optimize TNBS dose and concentration

Sixteen rats weighing 264.9 g (standard deviation [SD] ±14.5) were divided into two groups. Group 1 received 17.5 and group 2 received 22.5 mg TNBS (1 M, 293.17 mg/ml, product number 92822, Fluka, Buchs, Switzerland) diluted in 50% ethanol to a concentration of 60 mg/ml. The total volume instilled was ∼0.29 ml and ∼0.38 ml respectively for group 1 and 2. In a subsequent study, eight rats received TNBS in eight different doses (7–31.5 mg) diluted in 50% ethanol, to a total volume of 0.7 ml with concentrations ranging from 10–45 mg/ml.

TNBS was rectally instilled via a female urinary catheter (DCT Ch 10, Servoprax GmbH, Wesel, Germany). After removal of residual rectal fecal pellets, the catheter was advanced approximately to the splenic flexure. After instillation, the rats were held with the head down for one minute to prevent TNBS from leaking out. The colitis was evaluated with colonoscopy (Olympus uretero-renoscope, URF Type V) with picture documentation at Day 3, Day 7 and at Day 12. Biopsies were obtained using an Olympus biopsy forceps (FB-56 D-1, Olympus, Norway).

#### Main study

Eight female Sprague Dawley rats, age eight weeks and weighing 196.9 g (SD±14.3) were used. Based on the initial studies, TNBS dissolved in 50% ethanol to a concentration of 30 mg/ml in a total volume of 0.6 ml was instilled. Animal weights and clinical status were monitored throughout the study. Colonoscopy with photo documentation and tissue sampling was performed two days before (T0), and three (T3), seven (T7) and twelve (T12) days after induction of colitis. A modified Murine Endoscopic Index of Colitis Severity [30] (MEICS, 0–12, *Thickening of the colon* was not considered) was used for endoscopic evaluation and grading of the colitis. Five animals that developed similar endoscopically moderate colitis in the left colon were chosen for further studies.

During each colonoscopy, three biopsies were collected and snap frozen in liquid nitrogen for later RNA extraction. Another two biopsies were fixed in 4% buffered formaldehyde and then embedded in paraffin. The study was terminated at T12. The colon was divided longitudinally and one part fixed in formaldehyde for histologic examination and the other frozen in liquid nitrogen.

Animal weight data were compared using Student's t-test with equal variances after confirming this with f-test. The significance level was set at α = 0.05.

### RNA extraction, microarray amplification, hybridization, scanning and quantification

#### Rat mucosal samples

From each animal, three biopsies were collected at every time point in the main study (TNBS 30 mg/ml, 0.6 ml). Samples were pooled and RNA was extracted using the RNeasy Mini RNA extraction Kit (cat.no. 74106, Qiagen, Hilden, Germany) according to the manufacturer's protocol. Quality of extracted RNA was controlled using NanoDrop Spectrophotometer (Thermo Scientific, DE, USA) and Bioanalyzer (Agilent Technologies, CA, USA). Samples with RIN>7 were deemed suitable for downstream analysis. Biotinylated cRNA was prepared from 400 ng RNA for each sample using the Illumina TotalPrep RNA Amplification kit (Applied Biosystems/Ambion, Austin, TX, USA). Sample cRNA was subsequently hybridized on Illumina human RatRef-12 v1 expression BeadChips (Illumina, San Diego, CA, USA) and scanned on an Illumina BeadStation. Data from this analysis is publicly available at ArrayExpress, E-MTAB-1263.

#### Human mucosal samples

Gene expression profiles in mucosal samples from 25 patients with UC, 11 patients with CD, and 25 healthy controls were used for comparison with the TNBS-transcriptomes. The data used was drawn from a larger IBD gene expression analysis study performed at Norwegian University of Science and Technology/St. Olavs Hospital, Trondheim, Norway. All IBD samples were obtained from maximally inflamed colonic mucosa, while normal controls were taken from the hepatic flexure. Four endoscopic pinch biopsies were collected from each area. Three biopsies were immediately snap frozen in liquid nitrogen, while the remaining sample was fixed in 4% buffered formaldehyde. The formaldehyde-fixed samples were embedded in paraffin and 4 µm sections were cut and stained with hematoxylin-eosin for histological evaluation by an experienced pathologist. In cases where the evaluation differed from the macroscopic observations, samples were removed from the analysis. Frozen biopsies were homogenized using an Ultra-Turrax T 25 homogenizer (Zanke & Kunkel IKA-Laboratorie Technik, Staufen, Germany). Total RNA was extracted using Ambion *mir*Vana^TM^ miRNA Isolation Kit (Applied Biosystems, Foster City, CA, USA). RNA quantity, purity and integrity were assessed using a NanoDrop^TM^ Spectrophotometer (Thermo Scientific, Wilmington, DE, USA) and Bioanalyzer (Agilent Technologies, Santa Clara, CA, USA). Only samples with a RIN>7 were used in the subsequent microarray analysis. For each sample, 250 ng total RNA was used to generate biotinylated, amplified cRNA following the Illumina TotalPrer RNA Amplification Kit (Applied Biosystems/Ambion, Austin, TX, USA). The samples were hybridized on Illumina HT12 expression BeadChips (Illumina, San Diego, CA, USA) and scanned on an Illumina BeadStation. Informed written consent was obtained from all involved patients, and the study was approved by the Regional Medical Research Ethics Committee (approval no 5.2007.910). The study was registered in the Clinical Trials Protocol Registration System (identifier NCT00516776). Data from this analysis is publicly available at ArrayExpress, E-MTAB-184.

### Data preparation and bioinformatic analysis

#### Gene expression analysis

Raw data was exported from the Illumina GenomeStudio software and normalized using the *lumi* package for Bioconductor suite [31]. The data was quantile normalized and log2 transformed. Time course differential gene expression analysis was performed using the BETR package for R statistical environment [32]. Pairwise group comparisons were performed using a Student's t-test. The (FC) was used to express the changes in average gene expressions between studied groups. To standardize annotation across microarray platforms, Illumina probe identifiers were mapped to their corresponding Ensembl (accessed March 23, 2011) gene identifiers (IDs) [33].

Clusters of similar gene expression profiles were identified using the Affinity propagation (AP) algorithm [34], where dissimilarity was expressed as the negative Euclidian distance. Subsequently, gene clusters were enriched for over-represented Gene Ontology (GO) Biological Process (BP) terms [35] using the hypergeometric test. For a cluster with *n* genes and an *a priori* defined functional category with *K* genes, the hypergeometric test was used to evaluate the significance of overlap *k* between the cluster and GO-BP category [36], [37]. All *N* genes on a microarray were used as reference. To ensure specificity of annotation within a gene cluster, functional categories containing <5 or >1000 genes were removed.

### Comparison of TNBS to IBD transcriptomes

Concordance between TNBS-colitis and IBD transcriptomes was assessed at the level of individual gene loci as well as at the level of KEGG [38] and Reactome pathways [39]. To standardize comparisons between rat and human data, rat gene Ensembl IDs were mapped to respective human orthologs. In total, 6142 genes were considered. TNBS-colitis FCs were calculated by comparing gene expression profiles at T3, T7, and T12 to T0. Similarly, IBD FCs were computed by comparing CD and UC samples to normal (N) tissue. At the level of single gene loci, concordance tests were carried out by correlating FCs of the TNBS-colitis and IBD transcriptomes. Gene expression profiles with Student's t-test *p*<0.05 and absolute log2 FC ≥1.1 were considered differentially regulated.

To assess concordance at the level of biological pathways, gene expression FCs in TNBS-colitis and IBD transcriptomes were mapped to respective Reactome and KEGG pathways. To ensure that only well-characterized cascades were studied, pathways with fewer than 5 genes were excluded. Subsequently, a pathway-level FC was computed by averaging FCs of all genes that mapped to a specific pathway. Finally, TNBS-colitis and IBD pathway level FCs were correlated.

For all comparisons, Spearman's rho was used to estimate correlations between TNBS-colitis and IBD FCs. Respective *p*-values were computed for testing the hypothesis of no correlation against the alternative that there is a nonzero correlation and *p*<0.05 was called statistically significant. Analysis was performed using the Statistics toolbox for Matlab (2009a, The MathWorks, Natick, MA, USA).

### Candidate genes

Interleukin 1 alpha (IL-1α) and interleukin 1 beta (IL-1β) were chosen as general markers of inflammation. TLR2 and TLR4 were considered markers of bacterial regulation of inflammatory responses. In addition, based on the degree of regulation and statistical significance, genes encoding the peroxisome proliferator-activated receptor gamma (PPARγ) and the endogenous prion protein (PRNP [PrP^c^]) were further evaluated.

### Histological evaluation

Endoscopic biopsies and whole colon specimens were fixed in 4% buffered formaldehyde, placed in standard plastic briquettes for tissue specimens and processed via standard protocols (dehydration, clearing and paraffinization overnight). Sections from each block were cut in 4 µm thickness and stained with hematoxylin-eosin. The slides were examined by a surgical pathologist (ISN) and the degree and type of inflammation, ulceration and degree of regeneration/architectural distortion was assessed.

### In situ hybridization

In situ hybridization (ISH) on endoscopic biopsies was performed using RNA-probes for the following genes: IL-1α (NM_017019), IL-1β (NM_031512), TLR2 (NM_198769), TLR4 (NM_019178), PPARγ (NM_001145367) and PRNP (NM_012631) with the RNAscope® 2.0 assay kit (Advanced Cell Diagnostics Inc., Hayward CA, USA.), according to the manufacturer's protocol. Briefly, endoscopic biopsies were fixed in 4% buffered formaldehyde for five days and embedded in paraffin. Sections from each block were cut in 4 µm thickness and baked 1 hr at 60°C prior to use. After de-paraffinization and dehydration, the biopsies were air dried and treated with peroxidase blocker before gentle boiling in a pretreat solution for 15 min. Then, protease was applied for 30 min at 40°C. After pretreatment steps, the target probe was applied and hybridized for 2 hr at 40°C. Thereafter, the amplification steps including application of a horseradish peroxidase (HRP)-linked labeling probe were performed prior to DAB-visualization.

### PCR

RNA from 18 biopsies (no residual RNA was available from rat 2 and 4 at T3 and T7, respectively), was converted to cDNA with High Capacity RNA-to-cDNA Kit (Applied Biosystems, Foster City, CA, USA) according to the manufacturer's manual. PCR was performed using TaqMan® Array Plates (Life Technologies™, Carlsbad, CA, USA) on a Step One Plus™ Real Time PCR System. Genes with the largest FC alteration in the microarray data, serine peptidase inhibitor of the Kazal type 4 (SPINK4), adenosine deaminase (ADA), and fatty acid binding protein (FABP) 1 were analyzed. Actin beta (ACTB) was used as housekeeping gene. For each time point relative differential expression was calculated using the ddCT method followed by two-tailed paired t-test, T0 served as control.

## Results

### Optimization of the TNBS protocol

**Figure 1:**
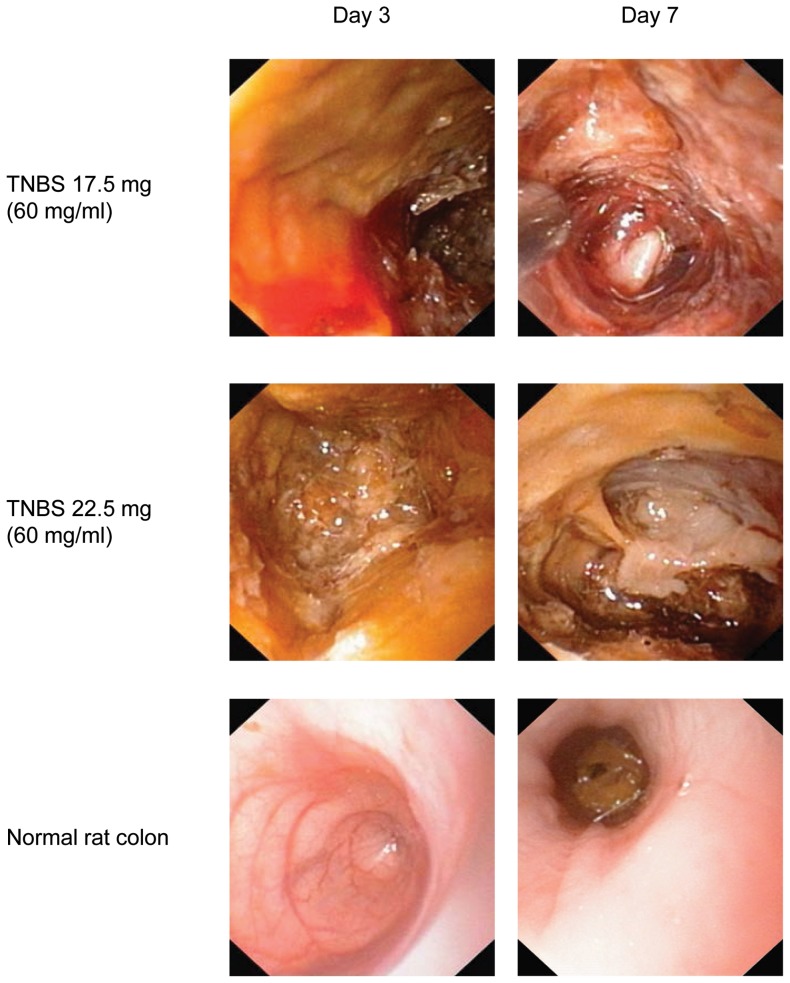
Endoscopic images demonstrating the effect of different doses of TNBS in a fixed concentration of 60 mg/ml. Severe colitis with a yellowish membrane and coprostasis was noted at day 3, and mucosal shedding, stenosis and necrosis was evident at day 7 in rats given TNBS 17.5 mg and 22.5 mg (0.29 ml and 0.38 ml, respectively). The bottom panel depicts examples of normal rat colon.

In order to standardize the TNBS-protocol to achieve a moderate colitis, we initially instilled TNBS in two doses (17.5 and 22.5 mg) at a fixed concentration of 60 mg/ml. The inflammation was severe in seven out of 16 rats in both treatment groups. MEICS-score ranged from 8–11 (9.9±0.9) at T7 (Figure 1). The study had to be terminated prematurely for animal welfare reasons.

**Figure 2:**
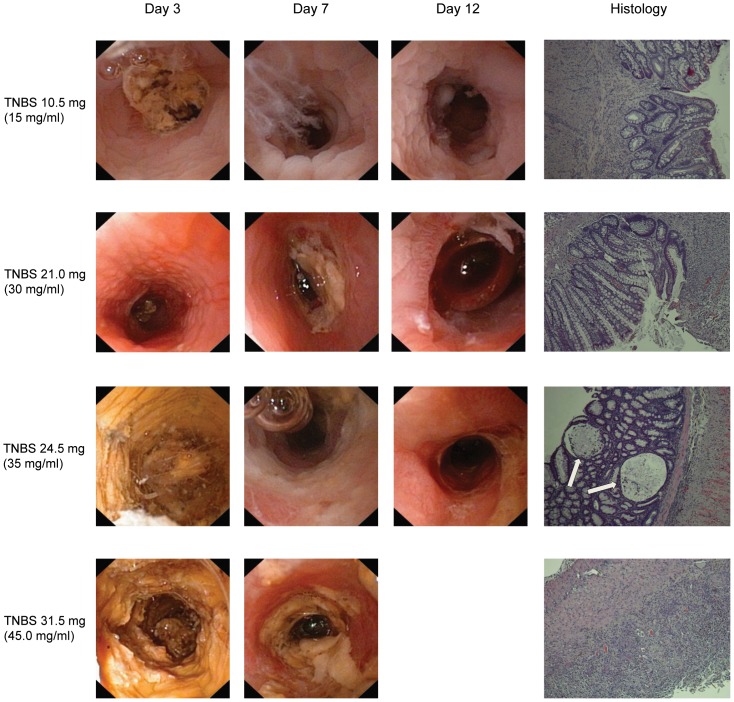
Endoscopic images demonstrating the effect of TNBS at different concentrations instilled rectally in a total volume of 0.7 ml. TNBS 10.5 mg (15 mg/ml) was associated with minimal mucosal inflammation and edema. TNBS 21.0 mg (30 mg/ml) resulted in an erythematous and edematous mucosa at day 3 and 7. At day 12 mucosal granulation and ulcerations were seen. TNBS 24.5 mg (35 mg/ml) and 31.5 mg (45 mg/ml) resulted in a more severe acute inflammation, and at day 7, larger areas of ulceration with fibrin cover were visible. At day 12, stenotic strictures developed in the rat receiving TNBS 24.5 mg. The rat receiving 31.5 mg was euthanized after the second endoscopy and consequently no endoscopic picture from day 12 can be shown. In the right column, histological pictures corresponding to mild, moderate and severe TNBS-colitis are included. TNBS 10.5 mg (15 mg/ml) resulted in only minimal inflammation with some architectural changes. TNBS 21.0 mg (30 mg/ml) resulted in ulceration that bordered the mucosa with inflammatory cell infiltration. In TNBS 24.5 mg (35 mg/ml), crypt distortion and abscesses (arrows) with mucosal and submucosal inflammatory infiltration were visible. Severe TNBS colitis is seen in the lower hitstologic picture with transmural inflammatory cell infiltration and total denudation of the mucosa. Objective x10 in all histological images.

The protocol was adjusted and TNBS was administered in different concentrations and a fixed volume of 0.7 ml. TNBS concentrations of 10 mg/ml and 15 mg/ml resulted in only slight endoscopic and histologic inflammation. Mucosal erythema and edema and histologic submucosal inflammation without ulcerations were seen at a TNBS concentration of 20 mg/ml. TNBS at 25–40 mg/ml resulted in mucosal edema and friability. Histologic examination revealed ulcerations and submucosal inflammation. TNBS 30 mg/ml induced a moderate inflammation with MEICS-score 7 and 5 at day 3 and day 7, respectively. The highest TNBS concentration (45 mg/ml) induced severe colitis with general weakening and the rat was therefore euthanized at day 7 (Figure 2).

The protocol optimization demonstrated that 0.7 ml of TNBS (30 mg/ml) induced a moderate colitis that lasted at least 12 days and declined between T7 and T12. However, a small fraction of TNBS tended to leak from the rectum immediately after instillation. The total volume was therefore reduced to 0.6 ml for the subsequent study.

### Characterization of moderate TNBS-colitis

**Figure 3:**
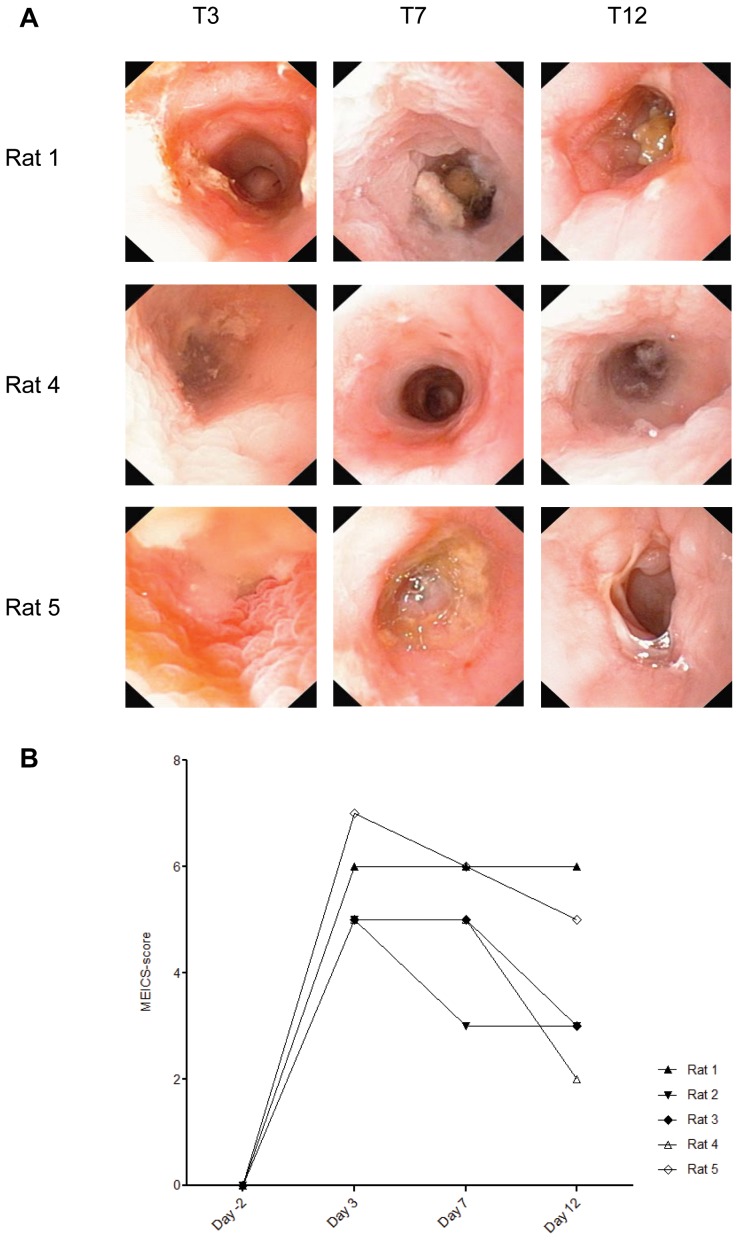
Representative endoscopic images of TNBS (30 mg/ml, 0.6 ml) associated changes at different time points and MEICS-score at T0, T3, T7 and T12. (A) Granulated and edematous mucosa at T3 and T7, and ulcerations at T12 were evident in Rat 1. At T7, small ulcerations/erosions were identified in Rat 4. In Rat 5, an ulceration is visible at T7 and at T12, a stricturing ulcer has developed. (B) The MEICS-score at T3 was significantly different from T12 (*p* = 0.02).

**Figure 4:**
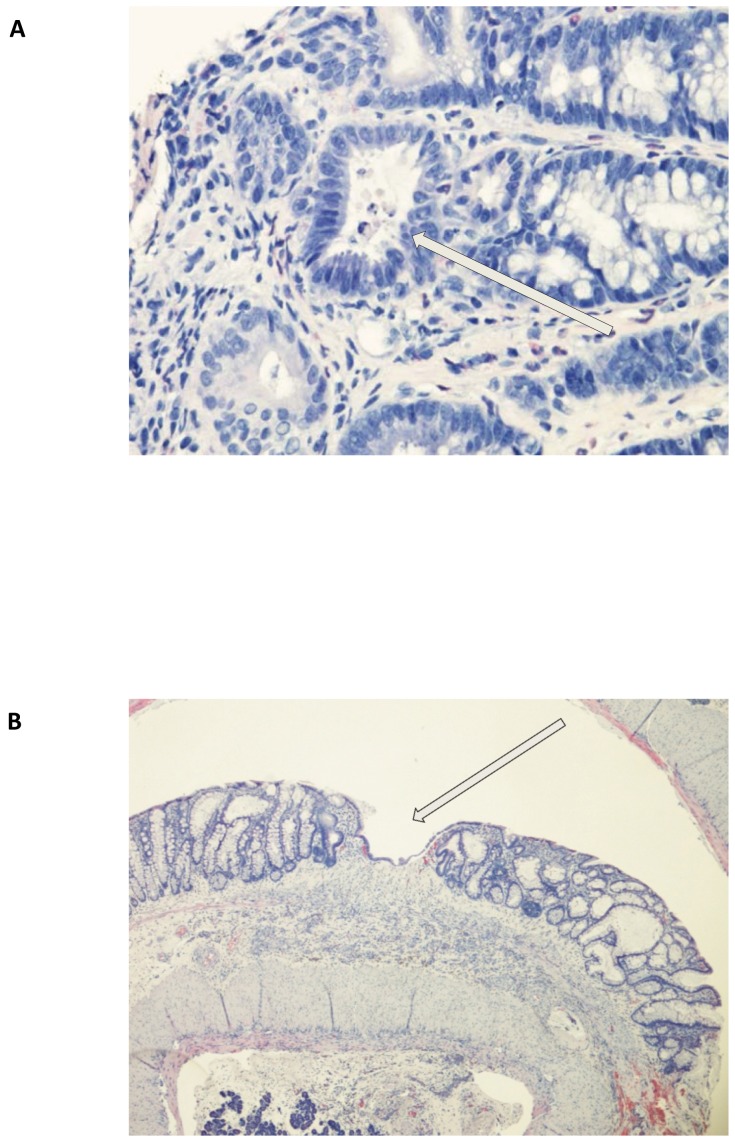
Histologic appearance of an endoscopic biopsy and whole colon specimen. (A) Endoscopic biopsy collected at T7 showing evidence of a crypt abscess (arrow), and mucosal gland distortion. Objective x40. (B) Histologic image of a whole colon specimen collected at termination of the study (T12) identifying distorted mucosal glands, and an ulceration with completed reepithelialization and underlying submucosal inflammation (arrow). Objective x4. The slides were stained with hematoxylin and eosin.

Five animals treated with TNBS 30 mg/ml (0.6 ml) developed moderate colitis from the splenic flexure down to the recto-sigmoid junction. The MEICS-score peaked at T3 (5.6±0.9, T3 vs. T12, *p* = 0.02) and T7 (5.0±1.2, T7 vs. T12, *p* = 0.11) (Figure 3A and B). An acute weight loss occurred at T3 (196.7±14.3 g at T0 versus 187.6±17.3 g at T3, mean weight loss 9.3 g, 4.7%, *p* = 0.003). The animal weights recovered to 200.4±24.7 g and 209.4±19.2 g at T7 and T12, respectively. Loose and bloody stools were noticed in two rats. Signs of cryptitis and architectural distortion were seen in endoscopic biopsies, whereas histologic examination of whole colon specimens at T12 demonstrated regenerative changes, ulcerations and transmural inflammation (Figure 4A and B).

### Temporal TNBS-colitis gene expression analysis

**Figure 5:**
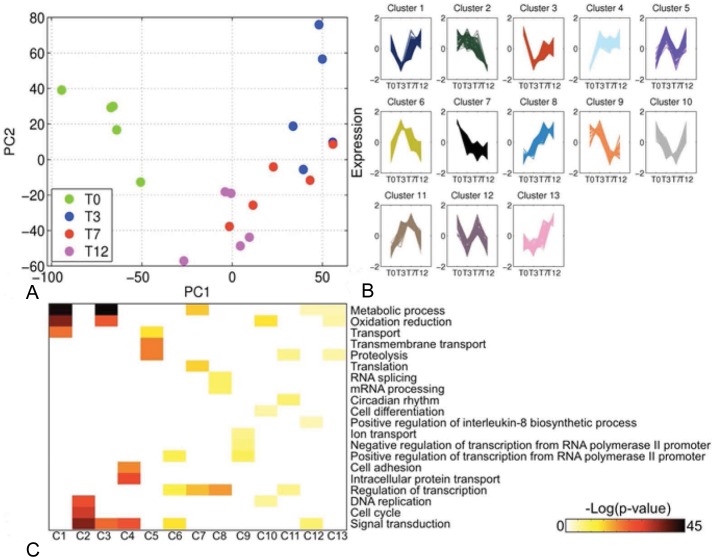
Differential expression analysis of the TNBS transcriptome. (A) Scatter plot of the Principal Component Analysis of 8316 genes in response to TNBS. (B) Expression profiles of genes assigned to the 13 clusters of similar expression (see Table S1). Only differentially expressed genes are included. (C) Heat map visualizing functional enrichment of the 13 clusters for over-represented Gene Ontology Biological Process terms. Darker shades represent a greater degree of enrichment.

Gene expression profiling of biopsies collected at time-points T0, T3, T7 and T12 was undertaken. After microarray preprocessing, expression profiles of 8316 genes were reduced to two Principal Components (PCs) to visualize differences in gene expression due to TNBS-induced inflammation (Figure 5A). Colitis induction resulted in distinct gene expression profiles while inter-individual temporal changes due to TNBS were not obvious.

To identify genes with sustained differential expression across all time points, the BETR algorithm was used. Genes with differential expression probabilities >0.99 were called significant. This analysis returned 3414 significantly regulated genes, which were subsequently clustered using the AP algorithm (Figure 5B). Significantly regulated genes were allocated to 13 clusters, with 588 and 63 genes assigned to the largest and smallest clusters, respectively. The full list of gene cluster assignments can be accessed in supplementary Table S1. Functional cluster enrichment for over-represented GO-BP terms identified processes such as *Oxidation reduction* (Clusters 1 and 3), *Cell cycle* (Cluster 2) and *Cell adhesion* (Cluster 4) (Figure 5C).

### Comparison of the TNBS and IBD transcriptomes

**Figure 6:**
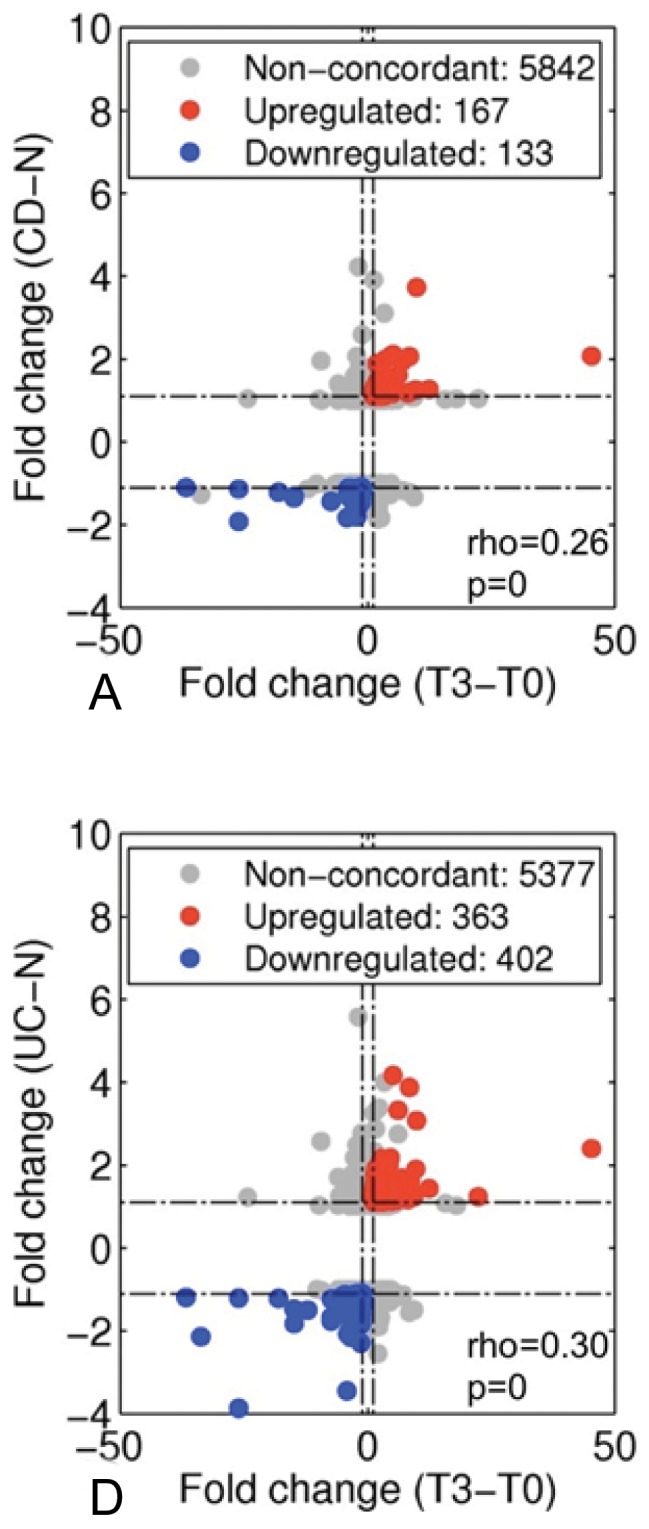
Concordance analysis between TNBS-colitis and IBD transcriptomes at the level of single gene loci. *T3 vs. T0*, *T7 vs. T0* and *T12 vs. T0* compared to *CD vs. normal* (top) and *UC vs. normal* (bottom). (A–F) Scatterplots of FCs in rat data (x-axis) compared to FCs in the human data (y-axis). Rho values correspond to Spearman correlation coefficients with p-values representing the level of significance of the correlation. Concordant up- and down-regulated genes are represented as red and blue circles respectively. Non-concordant genes are shown as grey circles. Numbers in the legend correspond to the total number of genes assigned to each category. B, C, E and F are only shown in the supplemental section (Figure S1).

To quantitate the similarity between the TNBS and IBD transcriptomes, differentially expressed genes at T3, T7 and T12 were compared to differentially expressed genes in CD and UC. Overall, 6142 rat genes could be mapped to human orthologs. The highest concordance was observed between CD and T3 (rho = 0.26, p = 0; *n* = 300 significantly regulated concordant genes) (Figure 6A). Similarly, the most concordance in differential expression was found between UC and T3 (rho = 0.30, p = 0; *n* = 765 significantly regulated concordant genes) (Figure 6D). Up-regulated genes in both TNBS-colitis and human IBD included ADA, PrP^c^, and IL-1α, while down-regulated genes consisted of aldehyde dehydrogenase 1 family, member A1 (ALDH1A1), and PPARγ. A full list of comparable gene changes in TNBS–colitis compared to CD and UC is available as supplementary Table S2.

**Figure 7:**
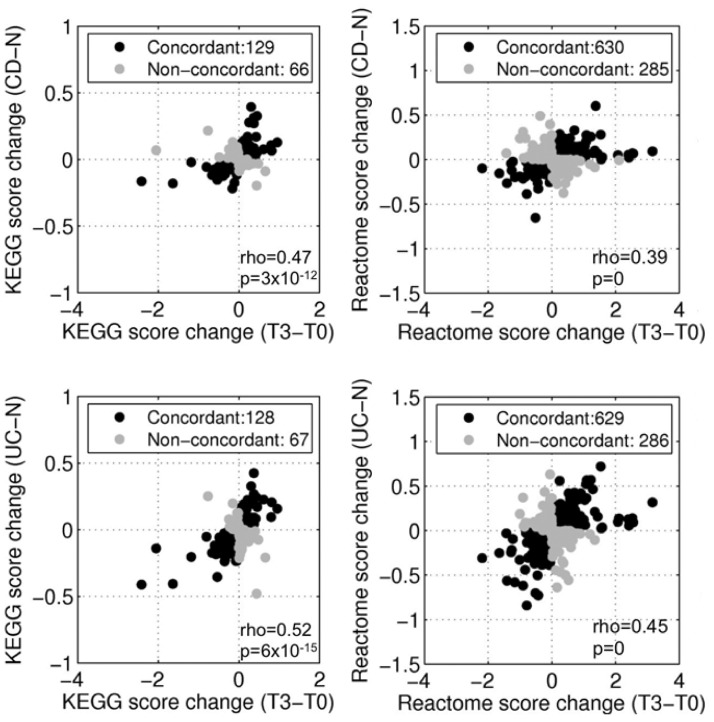
Concordance analysis between TNBS-colitis and IBD transcriptomes at the level of biological pathways. *T3 vs. T0* compared to *CD vs. normal* (top) *and UC vs. normal* (bottom). Left, scatter plots of pathway activity scores of KEGG pathways in TNBS and IBD samples. Right, scatter plots of pathway activity scores of Reactome pathways in TNBS and IBD samples. Rho values correspond to Spearman correlation coefficients with *p*-values representing the level of significance of the correlation. Numbers in the legend correspond to the total number of pathways assigned to each category. A similar figure (Figure S2) for concordant analysis of *T7 vs. T0* and *T12 vs. T0*, compared to *CD vs. normal* and *UC vs. normal* is shown in the supplementary section (KEGG and Reactome score change at the top and bottom, for T7 vs. T0 and T12 vs. T0, respectively).

As disruption in single gene expression can affect behavior of molecular pathways [40], concordance between the TNBS model and IBD was also assessed at the level of biological pathways. To ensure that results reflected a real phenomenon rather than a database-specific result, the FCs in gene expression were mapped to their respective identifiers in the KEGG (*n* = 195 pathways) and Reactome (*n* = 915 pathways) pathway databases. The highest concordance between CD and TNBS-colitis was observed at T3 (KEGG: Rho = 0.47, p = 3×10^−12^, Reactome: Rho = 0.39, p = 0) (Figure 7). Concordant pathways included Reactome pathways such as *Apoptosis*, *Cell junction organization*, *Interleukin-1 processing*; non-concordant pathways included *GABA-A receptor activation*, *Extrinsic pathways for apoptosis* and *Recycling of bile acids and salts*. Similarly, the most concordance was noted between UC and T3 (KEGG: rho = 0.52, p = 6×10^−15^, Reactome: rho = 0.45, p = 0) (Figure 7). For example, concordant Reactome pathways included *Nuclear receptor transcription pathway*, *TNF signaling*, and *VEGF ligand-receptor interactions*, while non-concordant Reactome pathways included *Apoptotic execution phase* and *TRAIL signaling*. The full list of regulated pathways in TNBS and IBD is accessible through supplementary Table S3.

### In situ hybridization (ISH) of target genes in TNBS-colitis

Markers of mucosal inflammation and selected microarray targets were analyzed using ISH. These included inflammatory cytokines (IL-1α and IL-1β) and TLRs (TLR2 and TLR4), as well as the computationally identified genes PPARγ and PRNP.

**Figure 8:**
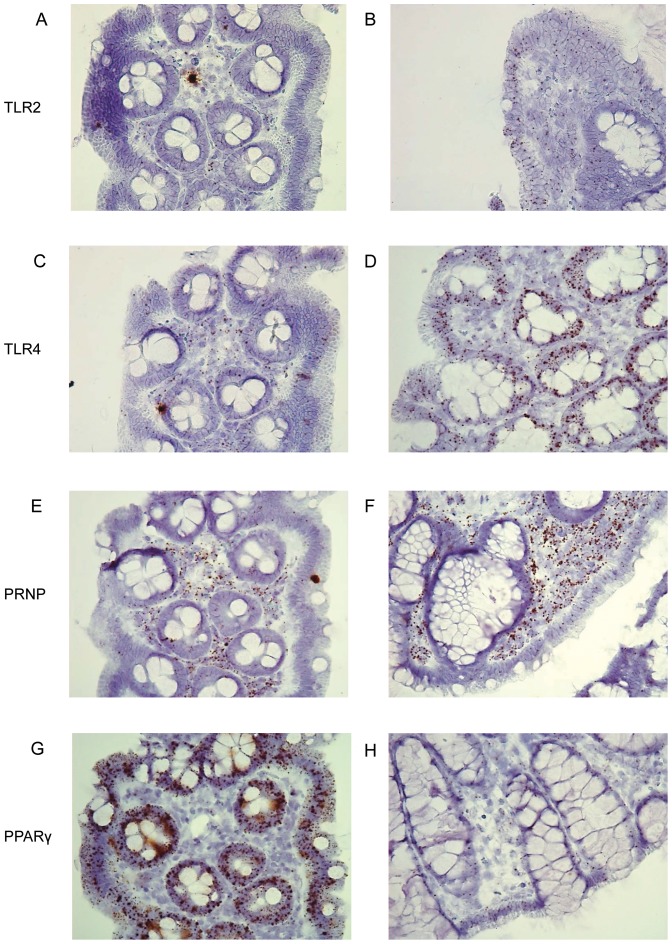
*In situ* hybridization (ISH) of significantly regulated genes in TNBS colitis mucosal biopsies. (A and C) The expression of TLR2 and TLR4 is scattered in the epithelium and submucosal immune cells at T0. (B and D) Increased TLR2 and TLR4 expression was noted in the epithelium at T7. (E) PRNP expression in submucosal immune cells at T0. (F) Intense PRNP expressing cells were evident in the submucosa at T12 and also to some degree in the epithelium. (G) Intense clusters of PPARγ expression in epithelial cells at T0. (H) At T12, the expression is almost abolished. Objective x40 in all pictures.

An overall increase in gene expression of IL-1α and IL-1β was evident in submucosal inflammatory infiltrates at T3-T12. However, changes in expression varied with the degree of inflammation in individual animals. Colitis induction resulted in increased expression of TLR2 and TLR4 in epithelial cells (Figure 8A, B, C and D). PRNP expression was increased both in submucosal inflammatory cells and to some degree also in epithelial cells (Figure 8F), while a substantial down-regulation of PPARγ was evident in the epithelium after induction of colitis (Figure 8H). The *in situ* results correlated well with the differential expression noted in the microarray, IL-1α (FC at T3 = 8.6, *p* = 0.008), IL-1β (FC at T7 = 4.7, *p* = 0.047), TLR2 (FC at T3 = 5.03, *p* = 0.005), TLR4 (FC at T3 = 2.09, *p*<0.001), PPARγ (FC at T12 = −4.6, *p*<0.001), and PRNP (FC at T3 = 2.1, *p* = 0.005). P-values and FCs for the target genes at every time point are provided in Table 1.

**Table 1:** P-values and FCs for the target genes at every time point.

Gene	T3		T7		T12	
	FC	P-value	FC	P-value	FC	P-value
IL-1α	8.63	0.008	3.38	0.091	1.58	0.209
IL-1β	6.16	0.063	4.74	0.047	1.94	0.071
TLR2	5.03	0.005	3.46	0.029	1.75	0.027
TLR4	2.09	<0.001	1.36	0.001	1.67	0.005
PRNP	2.13	0.005	2.11	0.005	1.89	0.016
PPARγ	−4.14	<0.001	−3.05	<0.001	−4.63	<0.001

### PCR validation of selected genes

**Figure 9:**
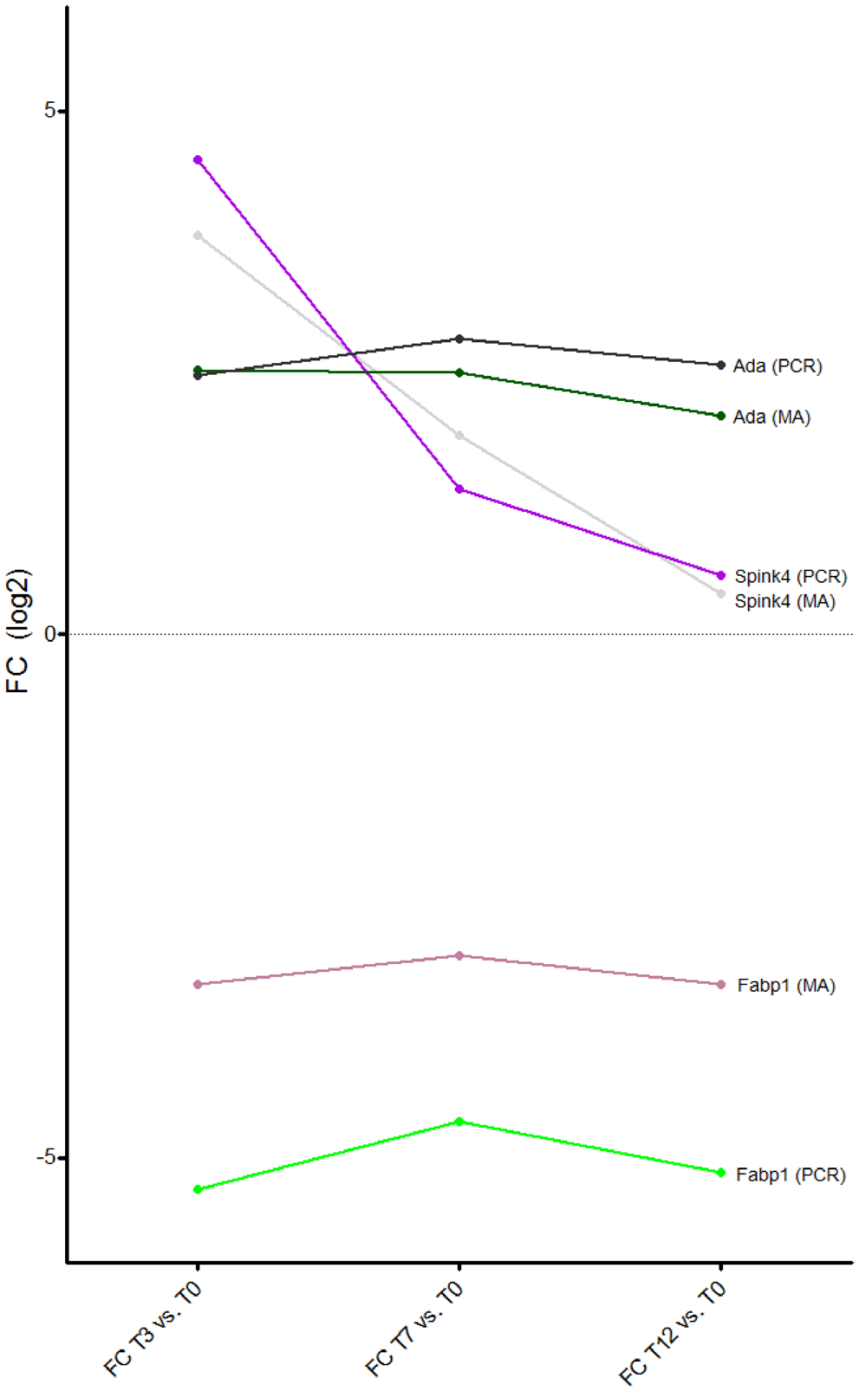
Graph displaying confirmatory PCR. Graph displaying FC for *T3 vs. T0*, *T7 vs. T0* and *T12 vs. T0* for SPINK4, ADA and FABP1 for both microarray (MA) and confirmatory PCR. FC is log2 transformed for illustrative purposes. All p-values are highly significant.

PCR was performed for the following genes: SPINK4, ADA, and FABP1. Mean FCs for SPINK4 were 93.26 for T3 vs. T0, 4.02 for T7 vs. T0 and 1.76 for T12 vs. T0. The corresponding FCs for ADA were 11.93, 16.87 and 13.20 and for FABP1 −199.96, −105.60 and −170.62 which were in accordance with the microarray results (Figure 9).

## Discussion

TNBS-colitis is widely used as a model for IBD. However, its resemblance to human disease has not been thoroughly explored. TNBS-colitis in rats was originally described as a model for induction of *long lasting inflammation and ulceration of the rat colon* [12], and the reproducibility was emphasized. The reproducibility and duration/chronicity of inflammation has, however, been subject to debate [14], [41]. In the current study, we standardized the protocol for induction of moderate TNBS-colitis in Sprague Dawley rats and evaluated temporal changes in gene expression profiles and biological pathways after the induction of colitis. Importantly, we quantitatively assessed concordance of the TNBS-colitis and IBD transcriptomes.

Doses and concentrations of TNBS used in previous studies differ markedly and no standardized protocol has been generally developed [14], [17]–[20]. We therefore aimed to optimize the method to achieve a reproducible moderate colitis. High concentrations of TNBS (60 mg/ml), administered in small volumes (0.29 and 0.38 ml) induced a localized severe inflammation resulting in deep colonic ulcerations, coprostasis, stenoses and ileus. Milder inflammation and more wide spread inflammation was achieved with lower TNBS concentrations in a larger total volume. A TNBS concentration of 30 mg/ml in a total volume of 0.6 ml resulted in a moderate inflammation involving most of the left colon. However, despite the use of a standardized protocol, a slight inter-individual dissimilarity in the extension and degree of inflammation was seen, reflected by variation in MEICS-score and expression of the pro-inflammatory cytokines IL-1α and IL-1β. Natural inter-individual variation and factors like anal leakage of TNBS and incomplete bowel emptying prior to the instillation are likely contributors to these observations. To minimize these effects, fecal pellets in the rectum should be eliminated prior to TNBS-instillation.

TNBS induces a hapten-mediated toxic inflammation, and consequently has unavoidable differences compared to IBD [42]. In addition to chemical inflammation, the intestinal microbiota is important in the development of TNBS-colitis [43]. Granulomas have been described in approximately 50% of rats with TNBS-colitis [12]. However, no granulomas were seen in the current study. This may reflect disparities in the microbiota seen in different animal housing facilities.

Endoscopic monitoring of TNBS-colitis with visual assessment has been described. However, in those studies no biopsies were obtained [44], [45]. In another study, transcriptomic analysis of TNBS-colitis was performed using whole colon specimens from animals sacrificed at different time points [21]. Consequently, temporal changes in gene expression in individual rats during colitis development has not been investigated. Furthermore, a comparison with a human IBD transcriptome has not been conducted. Our approach thus allows investigators to follow the development of colitis in individual animals visually, and collect biopsies for histologic evaluation and genetic analyses. Endoscopy three days after TNBS instillation allowed for a baseline evaluation of the inflammation. Rats with comparable colitis could therefore be identified prior to study inclusion. A similar approach has been described using MRI to grade the inflammation [14]. In addition, as the animals serve as their own controls it reduces the overall study number.

Computational analysis of longitudinal gene expression profiles in the TNBS-colitis model in this study revealed alterations such as a down-regulation of metabolism and up-regulation of tissue remodeling genes. This suggests that mucosal cells may be deprived of adequate energy sources as well as exposed to pro-fibrotic signaling cascades during the inflammatory response. Similar changes have been reported in IBD patients [46], [47] and models of experimental colitis [21]. Additionally, we noted down-regulation of processes including DNA replication and cell cycle, which involved genes such as caspase 1 and 3 (Casp1/3). Casp1 is an enzyme that proteolytically cleaves precursor forms of the inflammatory cytokines IL-1β and IL-18, while Casp3 proteolytically inactivates IL-33, which is a member of the IL-1 superfamily [48]. Interestingly, Casp^−/−^ mice are more susceptible to inflammation-induced tumorigenesis in the colon [49], while IL-33 was recently found to be increased in ulcerative colitis [50], [51]. Additionally, previous experimental models have demonstrated that Casp^−/−^ mice are also more susceptible to infections [52] and are more sensitive to DSS-colitis [53]. Taken together, the temporal analysis of TNBS-colitis gene expression profiles revealed biologically relevant changes in key IBD pathways, suggesting that the transcriptome dataset in this study is a useful resource for further biological discovery in this animal model.

Contributions of model organisms to our understanding of generalizable fundamental processes are irrefutable. However, little work has been done to quantitatively assess the concordance between TNBS-colitis and IBD transcriptomes. In our analyses, we noted a divergence between rat and human transcriptomes at the level of single gene loci with only 233–765 (3.8–12.5%) concordant gene loci out of 6142 analyzed. However, several key IBD genes were similarly regulated in the TNBS model and IBD. Genes involved in fibrogenesis and stricture formation [54], [55], including collagen type I alpha (COL1A1), matrix metallopeptidase 3 (MMP3) and TIMP metallopeptidase inhibitor 1 and 2 (TIMP1 and TIMP2) are examples thereof. These findings are consistent with other comparative assessments of human disease and animal models [56], [57]. Our group has shown that the REG family proteins and CXCL10 are up-regulated in IBD [58], [59]. The REG proteins probably play a role in induction of cellular proliferation and induction of apoptosis, thus being of importance for tissue repair and growth. CXCL10 seems to be released from epithelial cells acting as a ligand on CXCLR3+ T-lymphocytes, contributing to the inflammatory response in IBD. In contrast to these results, both REGIV and CXCL10 were down-regulated in the TNBS-model. It is possible that the duration of the TNBS-study was too short to detect a delayed activation of REGIV, however the discrepancy might also reflect the difference between IBD and TNBS-colitis. The discrepancy in regulation of CXCL10 also demonstrates the dissimilarity between human disease and TNBS-colitis. Other chemokines like CXCL1 and CXCL2, have previously been found up-regulated in TNBS-colitis [21] and were up-regulated in our data. We also found CXCL16 to be 1.5 fold elevated at T3. CXCL16 has a role in phagocytosis, mucosal defense and Th1-mediated inflammation, and has been implicated in the pathogenesis of IBD [60]–[62].

Despite differences at the level of single gene loci, considerably higher homology was noted between TNBS-colitis and IBD at the level of biological pathways. For KEGG-pathways 114–134 (58.5–68.7%) of 195 pathways, while for the Reactome 515–631 (56.3–69.0%) of 915 pathways were concordant. This result was expected, since previous work demonstrates that pathway level analysis is able to uncover subtle biological variability, which may not be captured by gene expression alone [63]. Concordant pathways included *Cell junction organization, Interleukin-1 processing*, and *Dysregulation of fatty acid metabolism.* These pathways represent key elements of IBD pathogenesis. For example, the ability to organize tight junctions, adherent junctions and desmosomes are important protective mechanisms that prevent microorganism invasion, mucosal damage and inflammation [64]. Similarly, aberrant regulation of IL-1α and IL-1β is a contributing mechanism in persistent inflammation in IBD [65], [66], while alterations in the expression of genes involved in fatty acid metabolism may contribute to the pathophysiology of ulcerative colitis [46].

Among non-concordant pathways, we identified *TRAIL signaling* as being different between the rat model and IBD. This signaling pathway mediates apoptosis and might be of importance for T-cell death as a therapeutic target in IBD [67].

Overall, these concordance analyses suggests that TNBS-colitis is an appropriate IBD model for the study of specific biological processes such as *Cell junction organization* and *Fatty acid metabolism*, while caution needs to be exercised when analysis is based on a single gene locus.

Other chemically induced colitis models exist. Colitis induced through addition of the polysaccharide dextran sodium sulfate (DSS) in the drinking water or rectal instillation of oxazolone are examples thereof [68]. DSS and TNBS have clinical similarities including bloody diarrhea and weight loss. However, inflammation in DDS seems to be caused by hyperosmotic damage and is confined to the mucosa and lamina propria, in contrast to the TNBS-model where inflammation is hapten-mediated and transmural [69]. Additionally germ free mice develop a lethal colitis when exposed to DSS [70], while a sterile colon seems to be protective against TNBS-colitis [43]. A comparison of genetic expression between DSS, TNBS and CD45RB transfer colitis mice showed that DSS and TNBS had 6 and 18 concordantly up- and down-regulated genes [71]. Another study found 152 and 22 concordantly up- and down-regulated genes between mice with DSS-colitis and UC from a total of 1609 differentially expressed genes [72]. In an analysis of cytokine patterns, closer resemblance was indicated between DSS-colitis and UC, while TNBS-colitis seemed closer to CD [73]. Oxazolone is another example of a rectally administered substance that causes a hapten-mediated colitis in the distal part of the colon with clinically similar symptoms as TNBS-colitis [68], [74]. Oxazolon-induced colitis is mainly driven by Th2 cytokines, while TNBS-colitis is Th1- and Th17-mediated [11], [73], [74].

Several gene knockout models in mice have been used to study possible pathogenetic mechanisms in IBD. Deletion of the regulatory sequence consisting of adenosine-uracil multimers (AU-rich elements [ARE]) in the 3′-untranslated region of cytokine encoding transcripts in mice (TNF-^ΔARE/+^), results in mice with increased transcription of TNF-α; after lipopolysaccharide (LPS)-stimulation these mice develop terminal ileitis and inflammatory arthritis [68], [75]. IL-10 knockout mice develop inflammation, mainly localized to the colon [76]. The IL-10 knockout model has highlighted the importance of IL-10 as an anti-inflammatory cytokine. IL-10 is increased in the serum of recovering IBD-patients [77]. IL-10 was not significantly regulated in our TNBS-model which might suggest that IL-10 does not participate in the healing of TNBS colitis.

We sought to validate key changes in the TNBS-colitis transcriptome using ISH. Genes with known roles and of potential importance in IBD were evaluated, namely IL-1α, IL-1β, TLR2, TLR4, PPARγ and PRNP. Gene expression profiling revealed that IL-1α and IL-1β were significantly up-regulated at T3 and T7, compared to T0, while TLR2, TLR4 and PRNP were significantly up-regulated at every time point (T3, T7 and T12) compared to T0. PPARγ was significantly down-regulated at every time point.

ISH confirmed that both IL-1α and IL-1β were weakly expressed in colonic epithelial cells while high expression was found in leukocytes and inflammatory infiltrates. Mediation of inflammation by IL-1α and IL-1β is balanced or counteracted via the IL-1 receptor antagonist (IL-1ra), which was significantly up-regulated at every time point. It has previously been shown in experimental colitis that neutralization of IL-1ra leads to more severe inflammation [78]. In concordance with the TNBS-colitis model, both IL-1α and IL-1β are elevated in IBD. An imbalance between these IL-1s and IL-1ra might contribute to the pathogenesis in IBD [65], [79].

The increased TLR2 and TLR4 expression seen in the transcriptome analysis was confirmed by ISH with dense staining in epithelial cells at the luminal surface and in the crypt epithelium. Hitherto, 10 TLRs have been described in humans [6]. TLR1, TLR2, TLR4, TLR5, TLR6, and TLR9 all recognize bacterial products or bacterial components [80]. While TLR2 and TLR4 were up-regulated, TLR5 was significantly down-regulated in our TNBS-colitis model. Intestinal homeostasis between tolerance to intestinal microflora and activation of an inflammatory response appears to be dependent on the type of TLR involved and whether basolateral or apical TLRs are activated [81]. Similar to the TNBS-colitis model, activation of both TLR2 and TLR4 is seen in IBD [5], [82], [83]. Probiotic treatment also activates TLR2, TLR4 and TLR9 and modulates cytokine production and secretion [84]. Interestingly, IL-10 and TLR4 double knockout mice have an accelerated colitis development compared to IL-10 and TLR9 double knockout and IL-10 single knockout which do not develop colitis [85]. TLR2 and TLR4 up-regulation in epithelial cells in this TNBS-colitis model as well as the cytokine up-regulation indicate bacterial activation of mucosal defense and inflammation [5]. Clearly, the mechanisms of TLR-response are complicated. The mechanisms for the balance of an adequate immune response and adaption of the innate immune system, on one hand, and an inadequate immune response, on the other hand, can partly be explained by tolerance development and polarization differences in TLR-receptor distribution [86], [87].

PRNP (PrP^c^) mRNA was significantly up-regulated in TNBS-colitis. This was verified by ISH which showed expression in submucosal immune cells and some expression in epithelial cells in animals not yet exposed to TNBS, but expression was more pronounced after colitis induction both in the submucosal immune cells and within the epithelium. PrP^c^ is expressed in several tissues, including neural tissue, gut-associated lymphoid tissue, enteroendocrine cells and enterocytes [28], [88]. Its function is not well known. Expression of PrP^c^ in the host is thought to be necessary for the propagation and transmission of prion infection [28]. In addition, both pro- and anti-inflammatory roles have been suggested. Reduced inflammatory infiltration has been seen in PrP^c^-deficient mice upon stimulation with TLR2 and TLR4 ligands [26]. In contrast, PrP^c^ over-expressing mice are resistant to colitis induction with DSS while PrP^c^ deficient mice had significantly higher levels of inflammatory cytokines compared to DSS-treated wild type mice [29]. Increased intestinal barrier permeability has been demonstrated in PrP^c^-deficient mice, further supporting a protecting role for PrP^c^ [89] in colitis and IBD.

PPARγ is one of three identified isoforms of peroxisome proliferator-activated receptors (PPARs) and is expressed in several tissues; expression is high in the colonic epithelial cells [90], [91]. It is thought to have an important role in modulation of inflammation. PPARγ suppresses inflammatory cytokines like TNFα, interleukin-6 (IL-6) and IL-1β [24] and maintains defensin production [25]. Gene expression analysis revealed down-regulation of PPARγ after colitis induction. In endoscopic biopsies evaluated by ISH, a high basal expression of PPARγ in non-inflamed colonic epithelium, while a considerable down-regulation in inflamed colon epithelium was identified. This was accompanied by down-regulation of FABP1. FABP1 transcription, in the microarray was confirmed by PCR. The intracellular level of FABP1 positively regulates PPARγ and the two proteins also have a direct protein-protein interaction [92]. Regulation of FABPs is seen in IBD [46], PPARγ is down-regulated in UC [93] and gene polymorphisms of PPARγ have been implicated in the pathogenesis of CD [42], [94]. PPARγ-agonists have been shown to ameliorate TNBS-induced colitis [95], [96] and the PPARγ ligand *rosiglitazone* has shown efficacy in mild to moderately active IBD [97]. PPARγ might also inhibit tumor growth in colorectal cancer [98]. Alternatively, FABP1 has been suggested as a biomarker of CD because a 10-fold up-regulation was found in one study [99]. Our findings, in contrast, are consistent with loss of PPARγ-mediated anti-inflammatory effects. This supports a potentially important function for PPARγ in the regulation of colonic inflammation and indicates that FABPs could also be a possible target for therapy.

Among the 20 genes with highest FC at all time points were SPINK4; lipopolysaccharide binding protein (LBP), ADA and RETNLB. PCR validation was performed for SPINK4 and ADA. SPINK4 has recently been identified as a risk locus for UC [100] and increased expression is seen in untreated celiac disease [101]. LBP transfers LPS to the LPS-signaling receptor complex (which also contains TLR4), promotes innate immunity against gram negative bacteria, and may serve as a marker of disease activity in CD [102]. ADA deactivates adenosine and thus diminishes the potentially protective effects of adenosine in mucosal inflammation and hypoxia. A beneficial effect after inhibition of ADA has been demonstrated in experimental colitis [103], [104]. RETNLB had a FC of 470 and 253 at T7 and T12, respectively. RETNLB-deficiency in mice increases intestinal permeability and RETNLB seems important to maintain intestinal homeostasis [105].

In conclusion, endoscopy with biopsies in TNBS-colitis models is useful to visually follow temporal changes of inflammation and obtain tissue for histologic and gene expression measurements. TLR and interleukin activation, PPARγ-inhibition and regulation of PRNP (PrP^c^), occurs in both TNBS-colitis and IBD. TNBS-colitis is an appropriate IBD-model to study specific biological processes like *TNF signaling*, *Cell junction organization*, and *Interleukin-1 processing*. We conclude that the TNBS-model may be suitable for studying agents targeting these pathways and provide translational information for clinical studies.

## Supporting Information

Figure S1 **Concordance analysis between TNBS-colitis and IBD transcriptomes at the level of single gene loci.** See Figure 6.
(TIF)

Figure S2 **Concordance analysis between TNBS-colitis and IBD transcriptomes at the level of biological pathways.** See Figure 7.
(TIF)

Table S1 **Genes identified with Ensemble IDs, Gene Symbols and Gene Names and their respective cluster assignments (1–13) with involved GO-BPs and KEGG Pathways.**
(XLS)

Table S2 **Concordant genes identified with Ensemble IDs, Gene Symbols and Gene Names with corresponding FC,** ***p*** **-value and False Discovery Rate (FDR) for TNBS, CD and UC, respectively.**
(XLS)

Table S3 **Homologous pathways (KEGG, Sheet 1) and (Reactome, Sheet 2) in TNBS-colitis compared to CD and UC for different time points of TNBS-colitis (T3, T7 and T12).**
(XLS)
