# Correction: Relevance of TNBS-Colitis in Rats: A Methodological Study with Endoscopic, Histologic and Transcriptomic Characterization and Correlation to IBD

**DOI:** 10.1371/annotation/af20399c-993e-436c-ad06-e0d8a657a7b4

**Published:** 2013-05-02

**Authors:** Øystein Brenna, Marianne W. Furnes, Ignat Drozdov, Atle van Beelen Granlund, Arnar Flatberg, Arne K. Sandvik, Rosalie T. M. Zwiggelaar, Ronald Mårvik, Ivar S. Nordrum, Mark Kidd, Björn I. Gustafsson

There was an error in the title of the article. "Historical" should be "Histologic" and "Transcripttomic" should be "Transcriptomic". The full, correct title is:

Relevance of TNBS-Colitis in Rats: A Methodological Study with Endoscopic, Histologic and Transcriptomic Characterization and Correlation to IBD

The correct citation is:

Brenna Ø, Furnes MW, Drozdov I, van Beelen Granlund A, Flatberg A, et al. (2013) Relevance of TNBS-Colitis in Rats: A Methodological Study with Endoscopic, Histologic and Transcriptomic Characterization and Correlation to IBD. PLoS ONE 8(1): e54543. doi:10.1371/journal.pone.0054543

