# Correction: Relevance of TNBS-Colitis in Rats: A Methodological Study with Endoscopic, Histologic and Transcriptomic Characterization and Correlation to IBD

**DOI:** 10.1371/journal.pone.0101382

**Published:** 2014-06-20

**Authors:** The PLOS ONE Staff

## Notice of Republication

This article was republished on June 3, 2014, to correct errors in the title that were introduced during the typesetting process. The publisher apologizes for these errors. The republication encompasses the errors addressed in the correction notice, published on May 2, 2013.

Please download this article again to view the correct version. The originally published, uncorrected article and the republished, corrected article are provided here for reference.

## Supporting Information

File S1 Originally published, uncorrected article.
(PDF)

File S2 Republished corrected article.
(PDF)
