# Supplementary figures and images for: Relevance of TNBS-Colitis in Rats: A Methodological Study with Endoscopic, Histologic and Transcriptomic Characterization and Correlation to IBD
Source: PLoS One. 2013 Jan 31;8(1):e54543. doi: 10.1371/journal.pone.0054543 (PMC3561356; doi:10.1371/journal.pone.0054543)

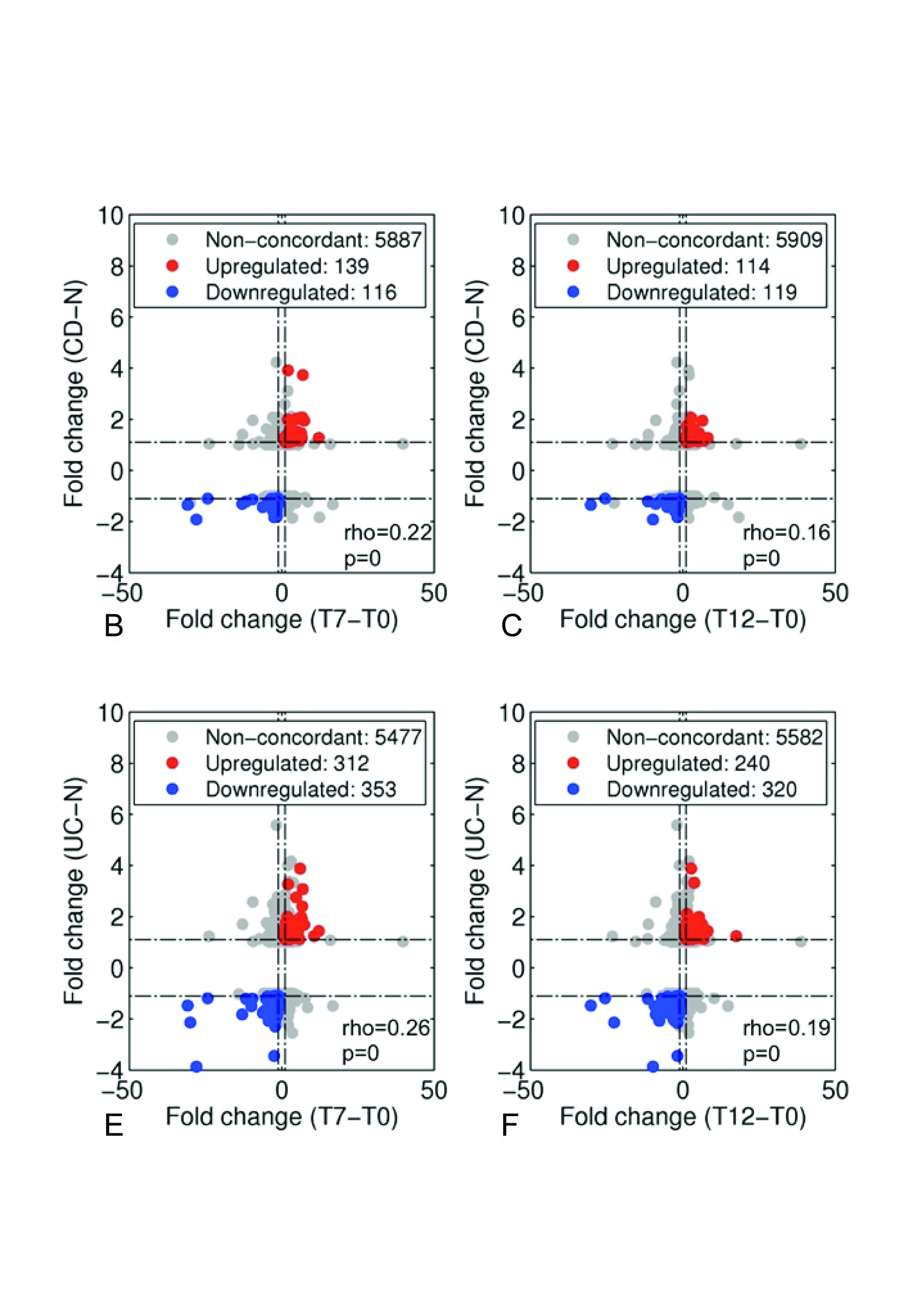

Supplement: Figure S1 — Concordance analysis between TNBS-colitis and IBD transcriptomes at the level of single gene loci. See Figure 6. (TIF) [file pone.0054543.s001.tif]

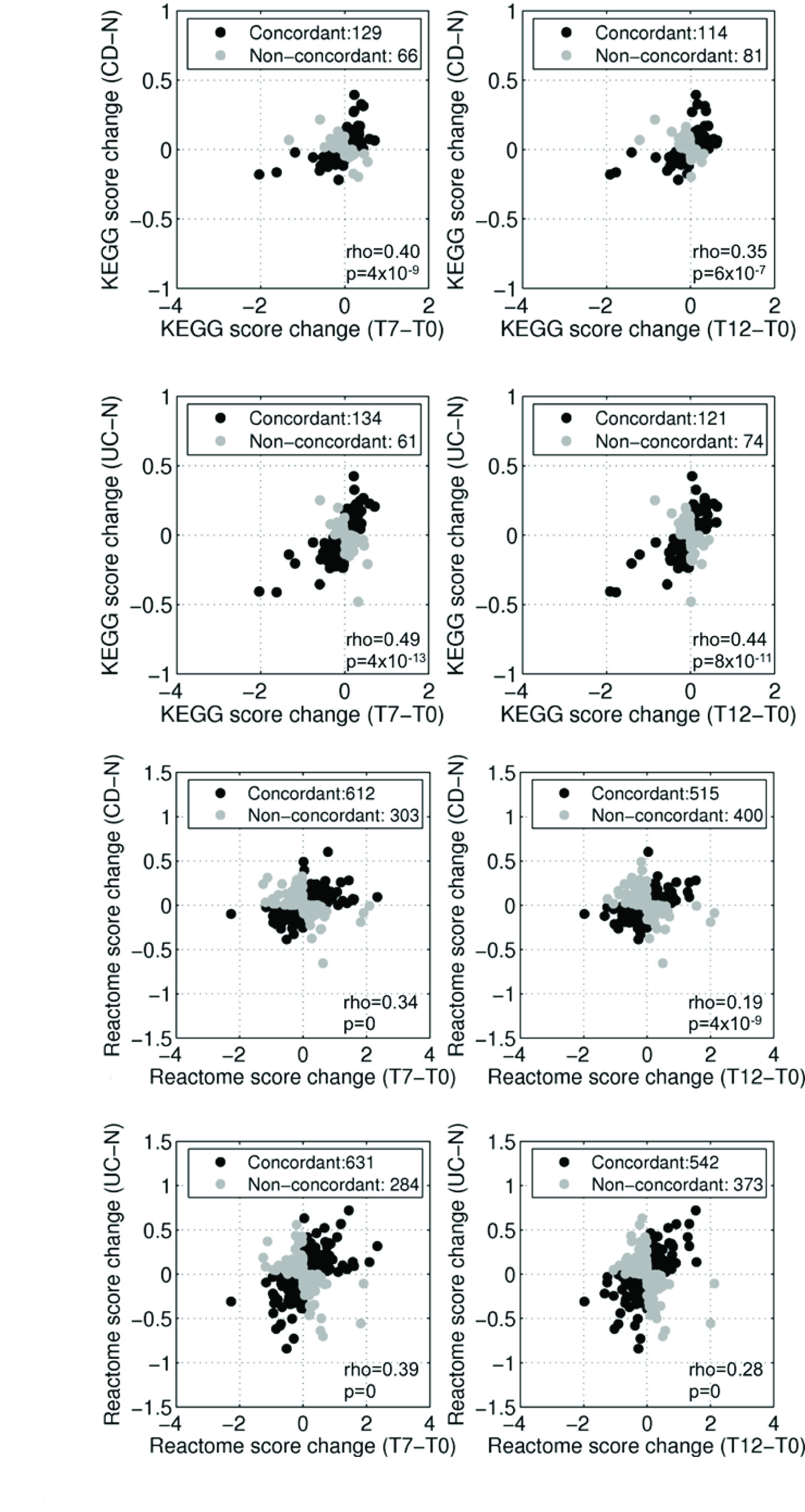

Supplement: Figure S2 — Concordance analysis between TNBS-colitis and IBD transcriptomes at the level of biological pathways. See Figure 7. (TIF) [file pone.0054543.s002.tif]
